# Cattle infection response network and its functional modules

**DOI:** 10.1186/s12865-017-0238-4

**Published:** 2018-01-05

**Authors:** Hamid Beiki, Abbas Pakdel, Ardeshir Nejati Javaremi, Ali Masoudi-Nejad, James M. Reecy

**Affiliations:** 1 Department of Animal Science, Iowa State University, Ames, IA 50011 USA; 2 Department of Animal Science, College of Agriculture, Isfahan University of Technology, Isfahan, 84156-83111 Iran; 3 Department of Animal Science, University College of Agriculture and Natural Resources, University of Tehran, Karaj, 31587-11167 Iran; 4 Laboratory of Systems Biology and Bioinformatics, Institute of Biochemistry and Biophysics, University of Tehran, Tehran, 31587-11167 Iran

**Keywords:** Bioinformatics, Data integration, Cattle, Immune response, Network analysis

## Abstract

**Background:**

Weighted Gene Co-expression Network analysis, a powerful technique used to extract co-expressed gene pattern from mRNA expression data, was constructed to infer common immune strategies used by cattle in response to five different bacterial species (*Escherichia coli, Mycobacterium avium, Mycobacterium bovis, Salmonella and Staphylococcus aureus*) and a protozoa (*Trypanosoma Congolense*) using 604 publicly available gene expression microarrays from 12 cattle infection experiments.

**Results:**

A total of 14,999 transcripts that were differentially expressed (DE) in at least three different infection experiments were consolidated into 15 modules that contained between 43 and 4441 transcripts. The high number of shared DE transcripts between the different types of infections indicated that there were potentially common immune strategies used in response to these infections. The number of transcripts in the identified modules varied in response to different infections. Fourteen modules showed a strong functional enrichment for specific GO/pathway terms related to “immune system process” (71%), “metabolic process” (71%), “growth and developmental process” (64%) and “signaling pathways” (50%), which demonstrated the close interconnection between these biological pathways in response to different infections. The largest module in the network had several over-represented GO/pathway terms related to different aspects of lipid metabolism and genes in this module were down-regulated for the most part during various infections. Significant negative correlations between this module’s eigengene values, three immune related modules in the network, and close interconnection between their hub genes, might indicate the potential co-regulation of these modules during different infections in bovine. In addition, the potential function of 93 genes with no functional annotation was inferred based on neighbor analysis and functional uniformity among associated genes. Several hypothetical genes were differentially expressed during experimental infections, which might indicate their important role in cattle response to different infections.

**Conclusions:**

We identified several biological pathways involved in immune response to different infections in cattle. These findings provide rich information for experimental biologists to design experiments, interpret experimental results, and develop novel hypothesis on immune response to different infections in cattle.

**Electronic supplementary material:**

The online version of this article (10.1186/s12865-017-0238-4) contains supplementary material, which is available to authorized users.

## Background

Cattle species provide a significant source of nutrition and livelihood to nearly 6.6 billion humans [1]. Bacterial infection accounts for significant production losses on cattle farms worldwide. For example, mammary gland infections alone are estimated to cost the United States dairy industry 2 billion dollars and are expected to have a similar impact in Europe [2]. In fact, immunity is an energetically costly process that requires a shift in energy away from nonessential functions to the immune system in the presence of pathogens [3–5]. Aside from the economic losses, bacterial diseases can impair animal welfare, and food-borne diseases can negatively impact human health [6]. For example in humans, salmonella infection is estimated to cause 3.8 million illnesses and 1.5 million deaths, annually [3]. Fundamental understanding of the host-pathogen interaction has a key role in the development of future strategies to mitigate these infections.

*Trypanosoma congolense*, is a parasite responsible for considerable cattle production losses and mortalityin Africa [4, 5]. In most cases, immunosuppression is the only immune response to this parasite which make the infected hosts susceptible to the secondary bacterial infections such as *Mycoplasma mycoides* and *Brucella abortus* [7]. Many experiments have been tried to explain the immune system reaction to this parasite [5], however it would be interesting to know the possible similarity between immune system response to this infection and different bacterial infections in cattle. Based on our knowledge this is the first study trying to address this issue.

Although mammalian species use specific strategies to respond to different infections, it can be hypothesized that some of these responses are common to many diverse infections [8]. This phenomenon has been reported in cattle immune response to infections with different strains of a given bacterial species [9, 10]. However, it is impractical to design an experiment to identify similar pathways across a diverse set of infections. In contrast, can insights be gleaned from meta-analyses of previously conducted infection studies? In plant species, there are efforts towards understanding these common processes [10–13]. Two recent studies [11, 12] in this area have used large collections of biotic stress related gene expression data in Arabidopsis to infer gene networks that predict common immune response strategies to different pathogens. Therefore, we hypothesized that a similar analysis in cattle could be informative.

A large collection of cattle infection gene expression data sets, that cover a wide range of tissues and developmental stages, was used to construct a large-scale bovine infection gene co-expression network. We have identified some putative gene functions and possible regulatory mechanisms. This effort will accelerate discovery of genes and lead to elucidation of the biological features responsible for immune response to infection in cattle.

## Results

**Fig. 1:**
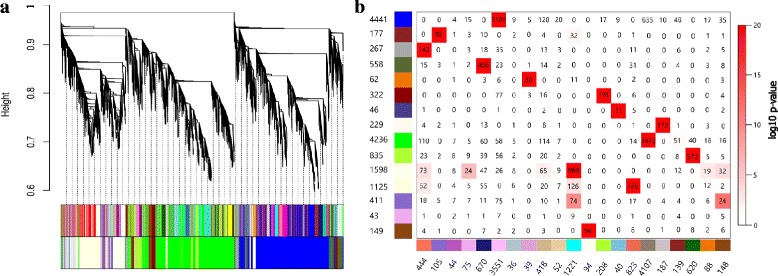
**a** Dendrogram of all differentially expressed transcripts clustered using the Topological Overlap Matrix dissimilarity measure. Each line of the dendrogram corresponds to a transcript. The first multi-color bar below the dendrogram shows the 50 pre-merged modules identified using the dynamic cutting method. The second multi-color bar shows the merged modules after permutation test of their eigengenes correlation (see Methods). **b** Correspondence of modules identified at %5 FDR (vertical axis) and 1% FDR rate (horizontal axis). Numbers in the table indicate gene counts in the intersection of the corresponding modules. Coloring of the table encodes -log (p), with p being the Fisher’s exact test *p*-value for the overlap of the two modules. The stronger the *red* color, the more significant the overlap is

**Fig. 2:**
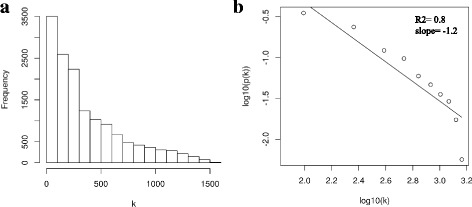
**a** Frequency of connectivity and **b** scale free topology evaluation of the constructed network

**Fig. 3:**
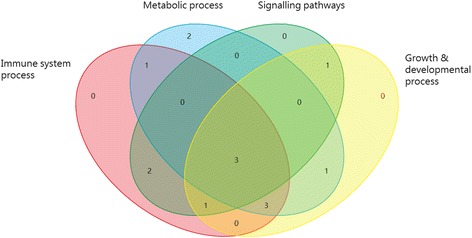
Venn diagram of modules functional classification

Eighty seven percent of transcripts in the Affymetrix Genechip Bovine Genome Array (14,499 of 16,607 transcripts) were differentially expressed (DE) in at least three different infection experiments: *Escherichia coli* (*E.coli*)*, Trypanosoma Congolense* (*T.congolense*)*, Mycobacterium avium* (*M.avium*)*, Mycobacterium bovis* (*M.bovis*)*, Salmonella* and *Staphylococcus aureus* (*S.areus*) (Additional file 1: Table S1). As shown in Table 1, the high number of shared DE transcripts between the different types of infections indicated that there were potentially common immune strategies used in response to these infections. These DE genes were grouped into 15 modules (groups of highly connected genes) using Weighted Gene Co-expression Network analysis (WGCNA) (Fig. 1a) [14]. The relatively high Scale-Free Topology Fitting Index indicated an approximate scale free topology in the network (Fig. 2). More than 90 % of modules (14 of 15 modules) showed high enrichment for GO/pathway terms related to “immune system process” (10 modules), “metabolic process” (10 modules), “growth and developmental process” (9 modules) and “signaling pathways” (7 modules) (Fig. 3 and Additional file 2: Table S2).

**Table 1:** The number of differentially expressed transcripts in response to different infections^a^

*E.coli*	14,429					
*M.avis*	8033	8072				
*M.bovis*	7577	6254	7614			
*S.areus*	8831	6178	5586	8862		
*Salmonella*	7730	5782	5451	7346	7759	
*T.congolense*	12,859	7475	7090	8515	7443	12,928
	*E.coli*	*M.avis*	*M.bovis*	*S.areus*	*salmonella*	*T.congolense*

^a^Diagonal elements represent the number of DE probe-sets in each infection type and off-diagonal elements represent the number of shared DE probe-sets between different infections

**Fig. 4:**
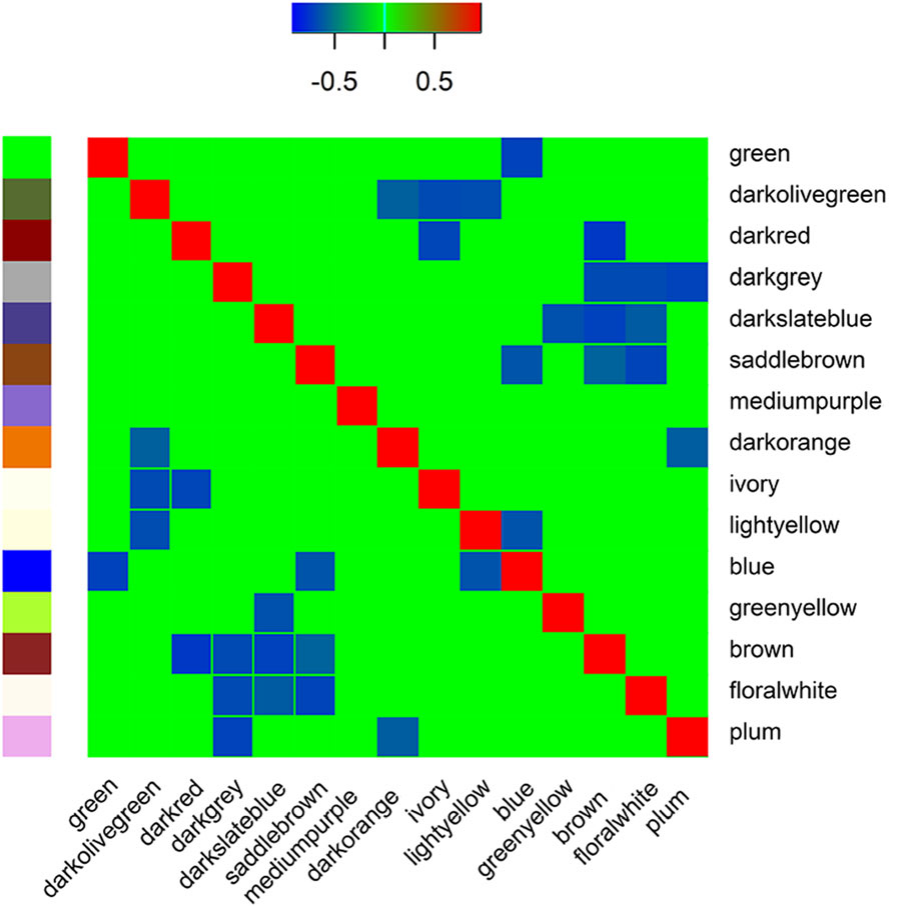
Heatmap representation of significant Spearman correlations between merged-modules eigengenes. Modules with significant positive correlation between their eigengenes have been previously merged (see Methods)

The largest module in the network, “blue” module, was highly enriched for several GO/pathway terms related to different aspects of lipid metabolism (Fig. 4 and Additional file 1: Table S1) There were significant negative correlations between eigengene values (see Methods) in this module and three other modules in the network (the “green”, “saddle brown” and “light yellow” modules) (Fig. 4). Altogether, these four modules contained 68% of all DE transcripts (9951 out of 14,499 transcripts) across experiments. The functionality of these related modules were examined in detail and are presented below.

**Fig. 5:**
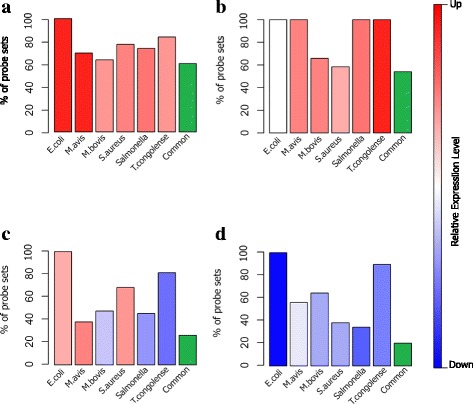
Proportion of DE transcript in response to different infections for the “*green*” (**a**), “*saddle brown*” (**b**), “*light yellow*” (**c**) and “*blue*” (**d**) modules. The *green* column in each graph shows the proportion of transcript that had DE in all infections. Relative expression levels are identified with colors

**Fig. 6:**
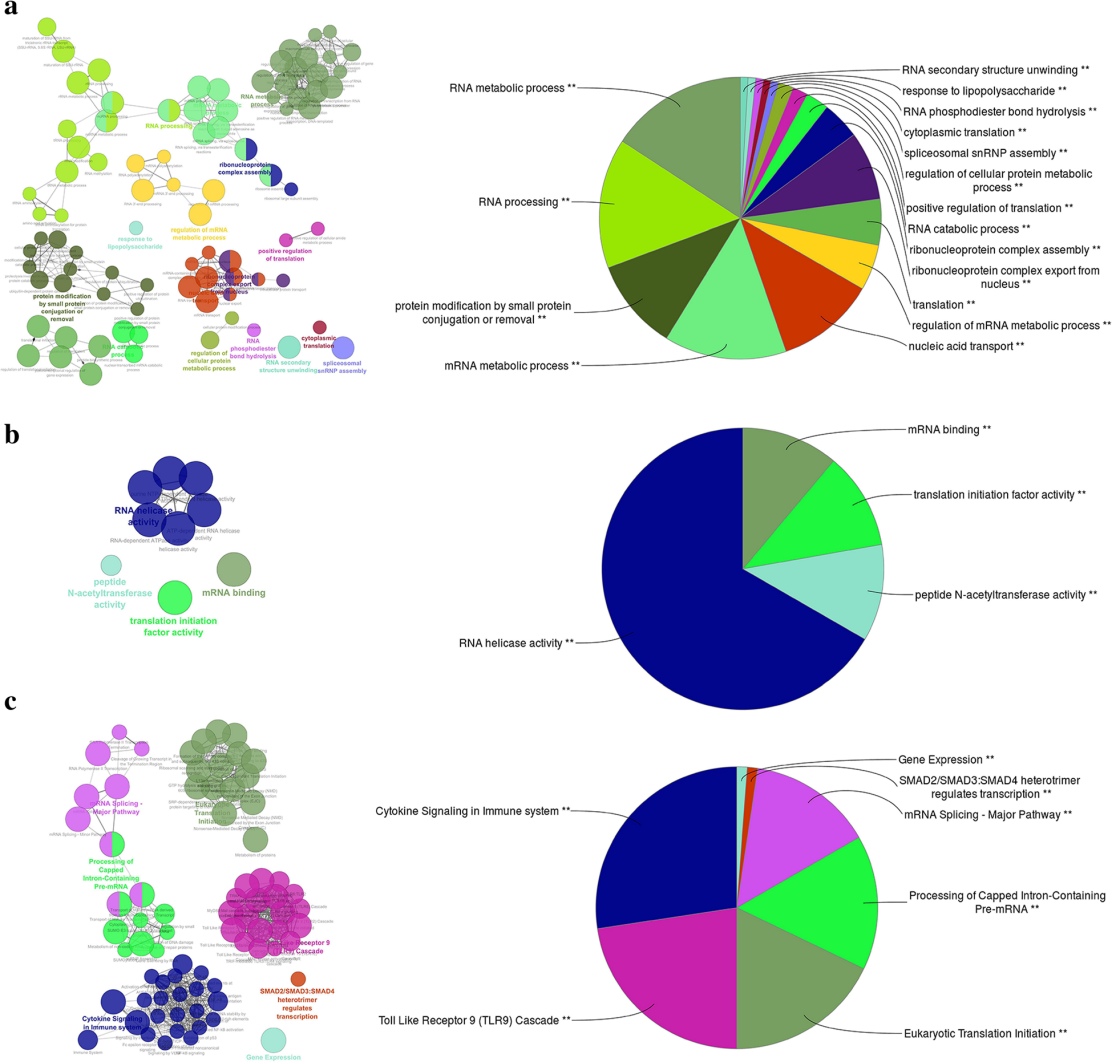
Functional analysis of the “*green*” module genes. Over-represented GO/pathway terms were grouped based on kappa statistics [62]. The size of each category within a pie chart represents the number of included terms. Only the most significant GO/ terms within groups were labeled. GO/pathway terms are represented as nodes, and the node size represents the term enrichment significance, while the edges represent significant similarity between categories. **a** Representative biological processes interactions among module genes. **b** Representative molecular function interactions among module genes. **c** Representative Reactome analysis interactions among module genes

The “green” module contained 4236 transcripts, which mapped to 3395 genes, with 354,062 edges. Sixty percent of these transcripts (2554 of 4236 transcripts) were DE in response to all six infection types (Additional file 2: Table S2). In addition, the expression level of DE transcripts was up regulated for the most part in response to these infections (Fig. 5a and Additional file 1: Table S1). Functional analysis of this module revealed high enrichment for several biological processes (BP) GO terms related to “regulation of gene expression” such as “RNA metabolic process”, “RNA processing” and “regulation of mRNA metabolic process” (Fig. 6a and Additional file 2: Table S2). Also, this module showed high enrichment for the BP GO term “response to lipopolysaccharide” which is the main component in the outer membrane of gram-negative bacteria. With respect to pathway analysis, this module was enriched for key immune regulatory pathways such as “cytokine signaling in Immune system”, “toll like receptor nine (TLR9) cascade” and “SMAD2/SMAD3:SMAD4 heterotrimers” which all regulate transcription (Fig. 6c and Additional file 2: Table S2). Molecular function of this module was related to “RNA helicase activity”, “mRNA binding”, “peptide N-acetyltransferase activity”, and “translation initiation factor activity” (Fig. 6b and Additional file 2: Table S2).

The “saddle brown” module contained 149 transcripts, which mapped to 138 genes, with 11,027 edges. All of the transcripts in the module were DE during infections with *E.coli, M.avium, Salmonella* and *T.congolense* (Fig. 5b and Additional file 2: Table S2). In addition, more than 50 % of these transcripts (79 of 149 transcripts) were DE in response to all six infections. With the exception of *E. coli*, the other five infection types resulted in up regulation of most of the genes in the module (Fig. 5b and Additional file 1: Table S1). This module exhibited several groups of enriched BP GO terms related to “positive regulation of apoptotic process”, “regulation of protein kinase B signaling” and “positive regulation of peptidase activity” (Additional file 2: Table S2 and Additional file 3: Figure S1A). With respect to pathway analysis, this module was highly enriched for “toll like receptor 4 (TLR4) cascade”, “signaling by NOTCH” and “deactivation of the beta-catenin transactivating complex” (Additional file 2: Table S2 and Additional file 3: Figure S1B).

The “light yellow” module contained 1125 transcripts, which mapped to 1021 genes, and had 24,532 edges. Most of these transcripts (769 of 1125 transcripts or 68 %) were DE in response to *E. coli* (1125 transcripts)*, S. aureus* (769 transcript) and *T. congolense* (915) infection (Fig. 5c and Additional file 1: Table S1). With the exception of *T. congolense,* the other two bacterial infections resulted in up regulation of most of the genes in the module (Fig. 5c and Additional file 1: Table S1). This module was highly enriched for BP GO terms related to “axon development” and “neuron development” processes (Additional file 2: Table S2 and Additional file 4: Figure S2A). With respect to pathway analysis, this module was enriched for pathways related to “class A/1 (Rhodopsin-like receptors)” and “GPCR downstream signaling” (Additional file 2: Table S2 and Additional file 4: Figure S2C). In accordance with these results, molecular function of this module was related to “cation channel activity” and “divalent inorganic cation transmembrane transporter activity” (Additional file 2: Table S2 and Additional file 4: Figure S2B).

The “blue” module contained 4441 transcripts, which mapped to 3577 genes, and had 725,412 edges. Most of these transcripts were DE during infections with *E. coli* (4441 transcripts)*, M. avium* (2856 transcript) and *T. congolense* (3978 transcripts) and in general their expression levels were down-regulated (Fig. 5d and Additional file 1: Table S1). This module exhibited several groups of enriched BP GO terms related to “fatty acid metabolic process”, “lipid oxidation” and “cholesterol metabolic process” (Additional file 2: Table S2 and Additional file 5: Figure S3A). In accordance with these results, this module was also enriched for the following pathway terms: “fatty acid”, “triacylglycerol”, “ketone body metabolism”, “bile acid and bile salt metabolism”, “metabolism of lipids and lipoproteins”, “cholesterol biosynthesis” and “peroxisomal lipid metabolism”(Additional file 2: Table S2 and Additional file 5: Figure S3C). Molecular function of this module was related to “iron ion binding” (Additional file 2: Table S2 and Additional file 5: Figure S3B).

### Hub gene analysis of four significantly connected modules

**Fig. 7:**
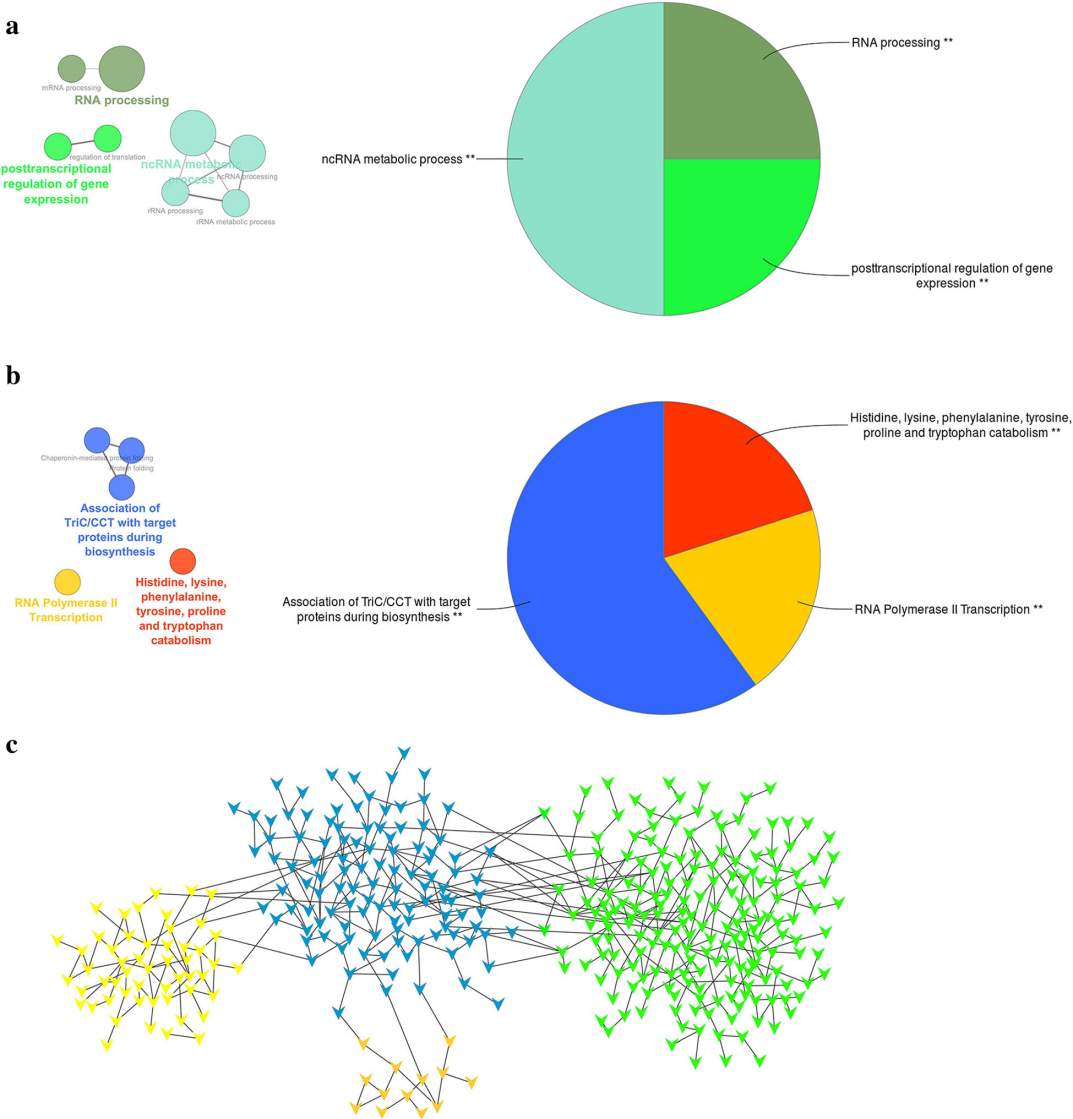
Functional analysis of the “*green*”, “*saddle brown*”, “*light yellow*” and “*blue*” modules hub genes. Over-represented GO/pathway terms were grouped based on kappa statistics [62]. The size of each category within a pie chart represents the number of included terms. Only the most significant GO/ terms within groups were labeled. GO/pathway terms are represented as nodes, and the node size represents the term enrichment significance, while the edges represent significant similarity between categories. **a** Representative biological processes interactions among genes. **b** Representative Reactome analysis interactions among hub genes. **c** Visualization of close interconnections (TOM > 0.1) between different modules hub genes in Cytoscape environment [63]

Three hundred and thirty-nine transcripts were identified as intra-modular hub nodes in the “green”, “blue”, “light yellow” and “saddle brown” modules. Of these transcripts, 44%, 36%, 16% and 4% of them belonged to the “green”, “blue”, “light yellow” and “saddle brown” modules, respectively (Additional file 6: Table S3). Functional enrichment analysis of these hub genes revealed their enrichment for gene regulatory activities such as BP GO terms related to “ncRNA metabolic process”, “posttranscriptional regulation of gene expression” and “RNA processing” (Fig. 7a) and pathways related to “RNA polymerase II transcription” and “association of TriC/CCT with target proteins during biosynthesis” (Fig. 7b).

Finally, the potential function of 93 genes, with no functional annotation, was inferred based on neighbor (genes that are highly connected to a given gene) analysis and functional uniformity among their associated genes (Additional file 7: Table S4). Running Blast for available sequences of these genes and known genes from different species revealed week sequence similarities. In addition, these hypothetical genes had several DE during experimental infections (Additional file 1: Table S1), which might indicate their important potential role in cattle response to different infections. The results of this study can be used as a new insight for possible biological function of these potential genes.

## Discussion

Infectious diseases result in large economic losses on cattle farms. Identification of genes and pathways involved in immune responses to these infections will ultimately result in new strategies to raise immunity against them. Based on the assumption of the existence common immune responses to different infection types, we constructed a cattle core infection stress response gene co-expression network using 604 arrays from 12 cattle infection experiments (Table 2). The consistent patterns of gene expression across several infection types found in this study (Fig. 5 and Additional file 1: Table S1) might point to the common immune response to these infections in cattle. However, because the size of the individual studies utilized in this meta-analysis, it is impractical to design an experiment to identify similar pathways across a diverse set of infections. Validation of these results are beyond the scope of this manuscript and require additional resources which are not available at this time. Also, it should be noted that these infections occurred at different locations and in animals with different genetic backgrounds. Caution should be used when extrapolating these findings across immune responses.

**Table 2:** Summary of datasets used in this study

Experiment	arrays^a^	Infection type^b^	Tissue^c^
E-MEXP-1778	120	*T.congolense*	Liver (80), Lymph (20), Spleen (20)
GSE24560	50	*E.coli* (26), *S.aureus* (24)	Mammary gland
GSE35185	41	*M.avium*	Macrophages
GSE25319	40	*E.coli*	Liver
GSE10695	27	*E.coli*	Mammary gland
GSE25413	24	*E.coli*	Mammary gland
GSE24217	23	*E.coli*	Mammary gland
E-TABM-878	23	*Salmonella*	Macrophages
GSE33309	21	*M.bovis*	Macrophages
GSE15025	15	*E.coli*	Mammary gland
GSE33359	8	*M.bovis*	Leukocytes
GSE50685	3	*E.coli*	Mammary gland

^a^Number of infected arrays using Affymetrix Genechip Bovine Genome Array platform

^b,c^Values in parenthesis correspond to the number of arrays for each experiment

To be more confident about the results, two networks were constructed based on DE transcripts that had been identified at either a 1% or 5% false discovery rate (FDR). Most of the modules identified at one FDR (e.g. 1% FDR) had a significant counterpart at the other FDR (5% FDR), which indicated the robustness of the constructed networks (Fig. 2b). We selected 5% FDR as a significant threshold in this study which allowed the capture of high confidence transcripts without a high false discovery rate.

The effectiveness of our approach is best illustrated by correspondence of these computational modules with actual biological entities. Most of the identified modules had over-represented GO/pathway terms, to “immune system process”, “growth and developmental process” and “signaling process” (Fig. 3), which indicated the potential interconnections between these processes in response to different types of infection in cattle.

Despite numerous studies in this area, the interaction between growth and immunity still remains largely unknown [15–17]. New evidence obtained from the model organism *Arabidopsis thaliana* points towards an indirect crosstalk between growth, signaling pathway and immune response [15]. In general, the growth-immunity trade-off can be explained by two hypotheses: first, considering the high energy demand of immunity and growth, the observed growth-immunity trade-off might reflect a competition for available energy resources and nutrients that are too limited to be allocated to both processes simultaneously [18, 19]. However, studies in model organisms have only been able to show weak correlations between these two processes [20, 21]. A more probable hypothesis is that biotic stress redirects cell cycle function from growth towards immunity using hormone-signaling activities [15, 16] thereby shifting the balance between these two functions. Mutual hormones in growth-immunity processes, such as Cytokines, have a key role in this cross-talk [15, 16].

Exposure to bacteria has a profound impact on mammalian cells metabolism and thereby can alter their cellular and molecular phenotypes and responses [22–25]. Our results demonstrate that the expression of a variety of lipid metabolism related genes (the “blue” module) are significantly down-regulated after infections with different bacterial species (Fig. 5d and Additional file 5: Figure S3). Alteration of lipid metabolism during bacterial infections has been reported in several experiments [22–26]. This cross-talk can be explained by three possible hypotheses. The first centers on the energy resources used by immune cells. This energy can be provided using aerobic glycolysis, or the oxidation of various substrates in the mitochondria (e.g. fatty acid beta-oxidation) [22]. Interconnections between these metabolic pathways and choices between them have high complexity and depend on various conditions; such as the relative availability of glucose, glutamine, and fatty acids, and whether there is sufficient oxygen to utilize oxidative phosphorylation [27]. In some cases cells preferentially use glycolysis for ATP generation even when oxygen is not a limiting, a process known as aerobic glycolysis or Warburg metabolism [22]. For example innate cells engage Warburg metabolism upon activation [22] of granulocytes [28] and dendritic cells [29], which are highly dependent on glucose for ATP production via aerobic glycolysis. The second hypothesis addresses the alternation of plasma membrane lipid composition to ensure adequate pathogen pattern recognition by immune cells [26, 30, 31]. Toll-like receptors (TLRs), which are a conserved family of pathogen associated pattern recognition molecules, are highly dependent on the membrane lipid composition for their functions [26, 30, 31]. Lastly, the third hypothesis considers the alteration of adipose tissue metabolism which participate in regulation of body energy metabolism [32] and host defense [33, 34]. Recently, Guo et al. [31] investigated the effect of lipopolysaccharide challenge on pigs white adipose tissue using proteomics techniques and reported significant suppression of lipid metabolism proteins (lipogenesis and lipolysis). Consistent with these findings, the expression level of key genes involved in lipid biosynthesis (ACACA, FASN, and SCD) and lipid beta-oxidation (ETFA, ETFB, ETFDH, GCDH and IVD) were down-regulated with all bacterial infections (Additional file 1: Table S1). These results might indicate the potential function of lipid metabolism genes in cattle response to bacteria.

The expression profile of each module can be summarized by one representative gene or eigengene [35, 36]. This can be defined as the first right-singular vector of the standardized module expression values as explained in [35]. There was a high inter-connection (TOM > 0.1) between hub genes in the “blue” module and three other module hub genes (Fig. 7c), which added to the significant correlation between their located module eigengenes (Fig. 4), which might indicate a potential functional relationship between these modules. For example, a TOM score > 0.1 meant that connection between two genes ranked in the top 99 percentile of connectivity across the network (Additional file 8: Figure S4). Among these three modules, two modules (“green” and “saddle brown”) had enriched GO/pathway terms directly related to “immune system process” (Fig. 6 and Additional file 3: Figure S1). The “saddle brown” module was also highly enriched for “regulation of fat cell differentiation” BP GO term (Additional file 3: Figure S1A). Three genes in this BP GO term (BMP2, SIRT1 and TCF7L2) had mutual regulatory functions in the immune system process and lipid metabolism based on their annotations [37]. Interestingly, there were close interconnections (TOM > 0.1) between these genes and PINK1, one of the “blue” module hub genes, which was involved in negative regulation of apoptotic process and positive regulation of release of cytochrome c from mitochondria based on its annotations [37].

The other module, the “light yellow” module, exhibited GO/pathway terms enrichment for neurogenesis process (Additional file 4: Figure S2). It has been shown that the immune system can directly or indirectly implicate neurogenesis via GPCRs signaling [38]. The regulation of lipid metabolism by the immune system can be imposed by growth factor cytokines and key activating receptors such as TLRs and G-protein-coupled receptors (GPCRs) [22]. Interestingly, these three immune related modules were highly enriched for signaling by TLRs (“green” and “saddle brown”), GPCRs (“light yellow”) and cytokines (“green”) pathways (Additional file 2: Table S2). In addition to these results, there were close inter-connections between the “blue” module hub genes and the other three module hubs (Additional file 5: Figure S3C). Functional enrichment analysis of hub genes revealed their high enrichment for GO/pathways related to gene regulatory activities (Fig. 7). These hubs were also enriched for the following pathway terms: histidine, lysine, phenylalanine, tyrosine, proline and tryptophan catabolism (Fig. 7b). Results from several studies indicate the important role of these amino acids in regulation of immune cells [39–43], lipid metabolism [44–46] and gene expression [39]. Altogether, these results might indicate the potential co-regulation of these modules.

## Conclusions

In summary, the results of this study provide rich information for experimental biologists to design experiments, interpret experimental results, and develop novel hypotheses on bovine immune response to bacterial infection. This effort will accelerate discovery of genes with important roles in response to different infection types and should lead to the elucidation of biological features responsible for immune response to these infections in cattle.

## Methods

### Gene expression data analysis

CEL files for the 607 publicly available Affymetrix Genechip Bovine Genome Arrays used in various infection experiments were downloaded from either National Center for Biotechnology Information Gene Expression Omnibus (NCBI GEO) [47] or European Bioinformatics Institute (EBI) ArrayExpress (December 2014) [48]. Arrays from individual experiments were normalized using the robust multichip analysis algorithm (RMA) for background adjustment with quantile normalization as implemented in the R affy package [49]. Quality tests were performed on the normalized array data using the Bioconductor arrayQualityMetrics package [50]. Arrays that failed all three outlier tests (i.e. Distances between arrays, Boxplots and MA plots) were excluded from further analyses. The final data set contained a total of 604 microarrays from 12 experiments, which comprised 395 infected samples (*E.coli, T.congolense, M.avium, M.bovis, Salmonella and S.aureus*) and 212 control samples (Table 2). Microarray probe-sets were mapped to *Bos taurus* UMD 3.1 genome assembly using AffyProbeMiner [51] with December 2014 release of *Bos taurus* genome annotation [52] as reference. Probe-set IDs with no mapped Entrez gene ID or Probe-set IDs that mapped to more than one Entrez gene ID were discarded. For each of the 12 data sets, batch information was obtained using the ‘scan date’ stored in the CEL files; chips run on different days were considered different batches and batch effects for each dataset were removed using the parametric ComBat algorithm [53].

The Bioconductor package Limma v. 3.22.7 [54] was used to identify differentially expressed probe-sets of infected versus normal control samples (45 comparisons) with a false discovery rate (FDR) equal to or less than 5%. The *P*-values were adjusted using the Benjamini-Hochberg procedure [55]. The fold-change values were calculated by subtracting normalized log2 transformed expression values of infected samples from healthy control samples. Only probe-sets with at least three biological replicates of the healthy control and infected samples were considered for analysis. To remove potential noise, all fold-change values associated with comparisons that were not considered significant by Limma were converted to ‘zero’, which in log2 scale corresponds to complete absence of differential regulation between treatments, as explained by Amrine et al. [11].

For simplicity, probe-sets equate to transcript throughout the manuscript.

### Weighted gene co-expression network analysis (WGCNA)

The WGCNA R package [14] was used to identify network modules from normalized gene expression values. Briefly, an adjacency matrix (correlation matrix) was formed with elements *r*_*ij*_, which were the Spearman correlation coefficient between expression values of probe-sets i and j. A connectivity measure (k) per probe-set was calculated by summing the connection strengths with other probe-sets. Subsequently as described by Zhao et al. [56], the adjacency matrix was replaced with the weighted adjacency matrix based on the β parameter with a scale-free topology structure (In a scale-free network, the frequency distribution of the connectivity, *p*(*k*), follows a power law distribution, *p*(*k*)~*k*^*β*^ [14]). The goodness of fit of the scale-free topology was evaluated by the Scale-Free Topology Fitting Index (*R*^2^), which was the square of the correlation between log(*p*(*k*)) (the frequency distribution of the connectivity in logarithmic scale) and log(*k*). A *β* coefficient of seven with Scale-Free Topology Fitting Index of 0.9 was used to develop a weighted adjacency matrix. The weighted adjacency metrix was used to then develop the topological overlap matrix (TOM) as described by Langfelder and Horvath [14]. The TOM reflects the relative interconnectivity between two genes based on their degree of shared neighbors across the whole network [14].

Dynamic Tree Cut algorithm [57], which utilized a gene tree dendrogram that was developed based on TOM-based dissimilarity (obtained by subtracting one from each element of the TOM matrix) using hclust algorithm [58], deep split was set to two and minimum module size was set to 25. The Dynamic Tree Cut may identify modules whose expression profiles were very similar. We explored the similarity between modules using modules eigengene values. The module eigengene was defined as the first right-singular vector of the standardized module expression values as explained in [35]. The multivariate permutation test was used to estimate the statistical significance of correlations using the following procedure: In each permutation run, the order of module i eigengene was held constant and the eigengenes of the other modules were randomized for 10,000 permutations. Spearman correlations between module eigengenes were calculated in each run of permutation and the empirical probability distribution of the randomized correlations for each pair of modules were used to define significant correlations using a *p*-value threshold of 0.05. After finding significant correlations, un-significant correlations were changed to “zero” and similar modules were merged using mergeCloseModules and height cut of 0.4 (correspond to the minimum positive significant correlation, i.e. 0.6). All other WGCNA parameters remained at their default settings.

In each module, a transcript was considered as an intra-modular hub node if its connectivity was equal or more than 90% of the most connected node.

### Modules functional analysis

ClueGO [59], a widely used Cytoscape plugin, was used to identify potential biological interpretation of functional modules in the network. The latest updates of gene ontology annotation database (GOA) [37] and Reactome pathway database [60] (released November 2015) were used in these analyses. Genes included in Affymetrix Genechip Bovine Genome Array were used as reference. Ontologies were designated as biological processes, molecular function and Reactome pathways. The GO tree interval ranged from three to 20 with the minimum number of genes per cluster set to three. Term enrichment was tested with a right-sided hyper-geometric test that was corrected for multiple testing as described by Benjnamini-Hochberg [61]. Only pathways that were significantly enriched (*p* ≤ 0.05) were included in the analysis. Kappa statistics were used to link and group the enriched terms and functional grouping of them as described in [59]. The minimum connectivity of the pathway network (kappa score) was set to 0.4 units.

## Additional files

Additional file 1: Table S1. Module membership, connectivity and expression pattern of 14,499 probe-sets used in this study. (XLSX 1290 kb)

Additional file 2: Table S2. Over-represented GO/Pathway terms in the co-expressed modules. (XLSX 206 kb)

Additional file 3: Figure S1. Functional analysis of the “saddle brown” module genes. Over-represented GO/pathway terms were grouped based on kappa statistics [62]. The size of each category within a pie chart represents the number of included terms. Only the most significant GO/ terms within groups were labeled. GO/pathway terms are represented as nodes, and the node size represents the term enrichment significance, while the edges represent significant similarity between categories. (A) Representative biological processes interactions among module genes. (B) Representative Reactome analysis interactions among module genes. (PDF 1302 kb)

Additional file 4: Figure S2. Functional analysis of the “light yellow” module genes. Over-represented GO/pathway terms were grouped based on kappa statistics [62]. The size of each category within a pie chart represents the number of included terms. Only the most significant GO/ terms within groups were labeled. GO/pathway terms are represented as nodes, and the node size represents the term enrichment significance, while the edges represent significant similarity between categories. (A) Representative biological processes interactions among module genes. (B) Representative molecular function interactions among module genes. (C) Representative Reactome analysis interactions among module genes. (PDF 1482 kb)

Additional file 5: Figure S3. Functional analysis of the “blue” module genes. Over-represented GO/pathway terms were grouped based on kappa statistics [62]. The size of each category within a pie chart represents the number of included terms. Only the most significant GO/ terms within groups were labeled. GO/pathway terms are represented as nodes, and the node size represents the term enrichment significance, while the edges represent significant similarity between categories. (A) Representative biological processes interactions among module genes. (B) Representative molecular function interactions among module genes. (C) Representative Reactome analysis interactions among module genes. (PDF 2680 kb)

Additional file 6: Table S3. Over-represented GO/Pathway terms in the “green”, “saddle brown”, “light yellow” and “blue” module hub genes. (XLSX 43 kb)

Additional file 7: Table S4. Functional enrichment analysis of close neighbors (TOM > 0. 1) of 85 un-annotated genes in the network. (XLSX 50 kb)

Additional file 8: Figure S4. Frequency of TOM connectivity in the network. (PDF 262 kb)

## Abbreviations

ATP: Adenosine triphosphate
BP: Biological process
DE: Differential expression
EBI: European Bioinformatics Institute
FDR: False discovery rate
GEO: Gene expression omnibus
GO: Gene ontology
GOA: Gene ontology annotation database
GPCRs: G-protein-coupled receptors
NCBI: National Center for Biotechnology Information
ncRNA: non-coding RNA
*R*^2^: Scale topology fitting index
TOM: Topological overlap measure
WGCNA: Weighted Gene Co-expression Network Analysis
