# Supplementary figures and images for: Cattle infection response network and its functional modules
Source: BMC Immunol. 2018 Jan 5;19:2. doi: 10.1186/s12865-017-0238-4 (PMC5755453; doi:10.1186/s12865-017-0238-4)

A

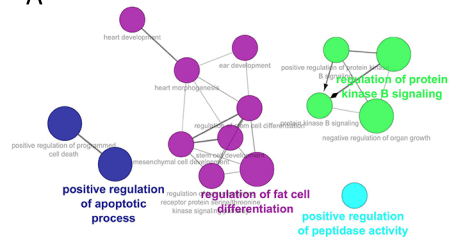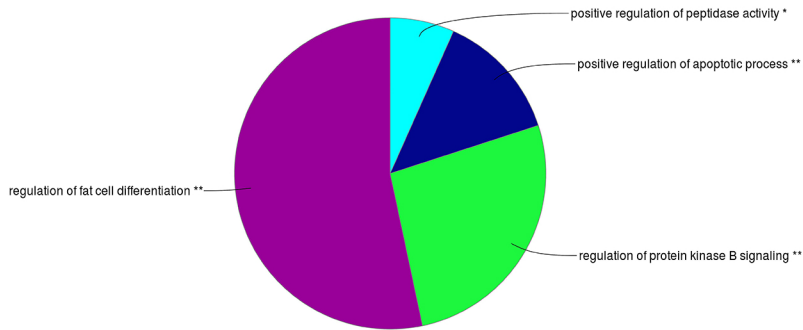

B

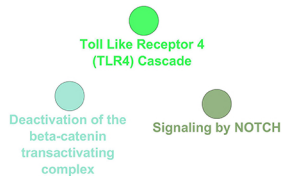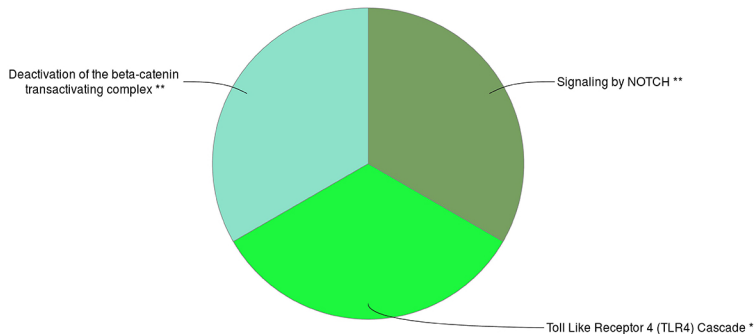

Supplement: Supplementary file 3 — Functional analysis of the “saddle brown” module genes. Over-represented GO/pathway terms were grouped based on kappa statistics [62]. The size of each category within a pie chart represents the number of included terms. Only the most significant GO/ terms within groups were labeled. GO/pathway terms are represented as nodes, and the node size represents the term enrichment significance, while the edges represent significant similarity between categories. (A) Representative biological processes interactions among module genes. (B) Representative Reactome analysis interactions among module genes. (PDF 1302 kb) [file 12865_2017_238_MOESM3_ESM.pdf]

A

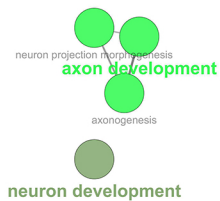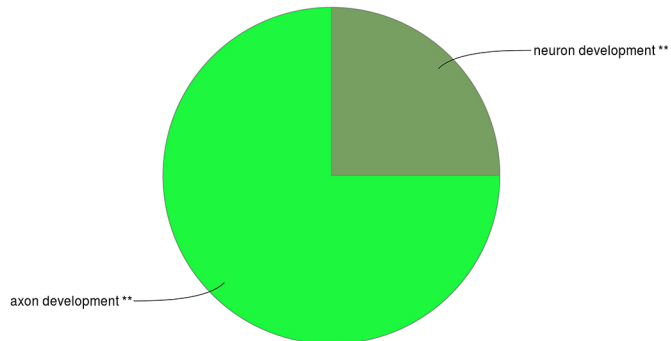

B

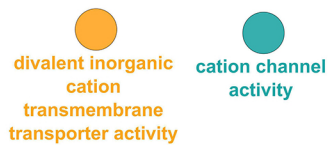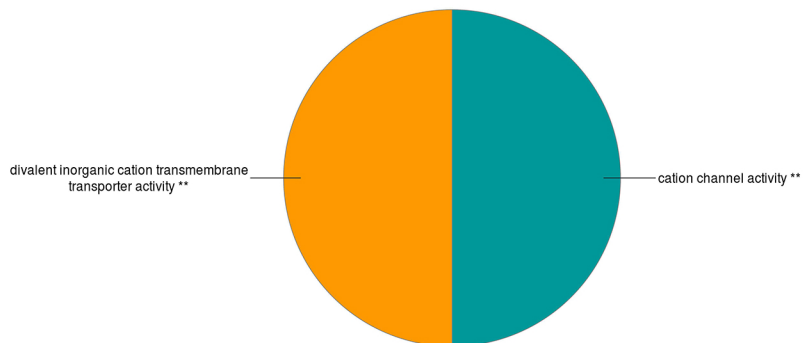

C

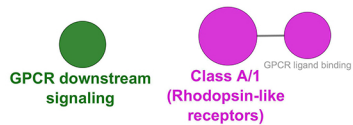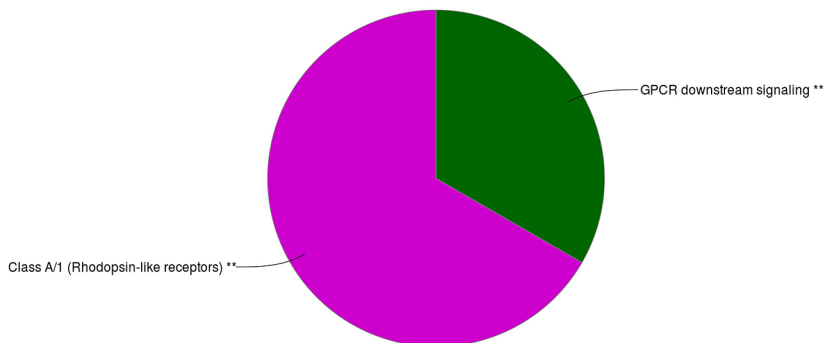

Supplement: Supplementary file 4 — Functional analysis of the “light yellow” module genes. Over-represented GO/pathway terms were grouped based on kappa statistics [62]. The size of each category within a pie chart represents the number of included terms. Only the most significant GO/ terms within groups were labeled. GO/pathway terms are represented as nodes, and the node size represents the term enrichment significance, while the edges represent significant similarity between categories. (A) Representative biological processes interactions among module genes. (B) Representative molecular function interactions among module genes. (C) Representative Reactome analysis interactions among module genes. (PDF 1482 kb) [file 12865_2017_238_MOESM4_ESM.pdf]

A

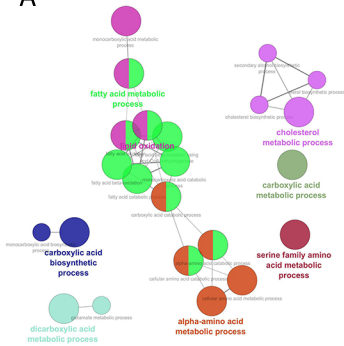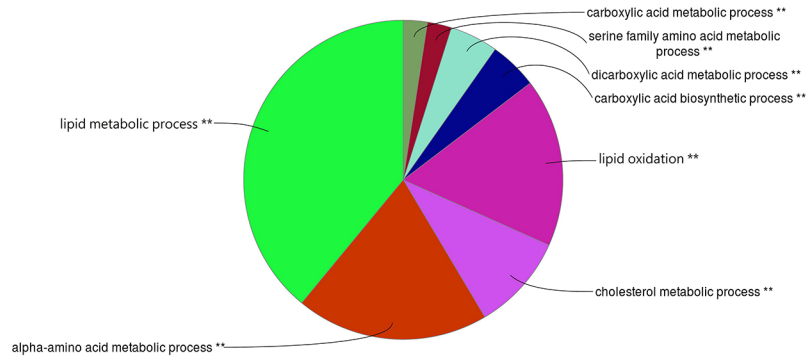

B

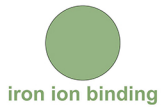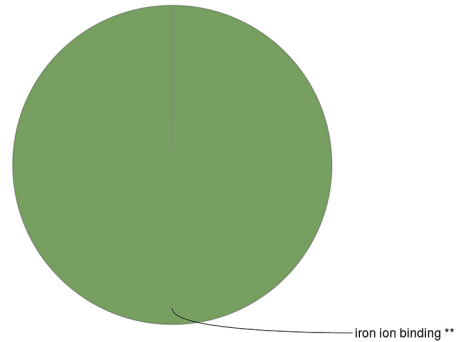

C

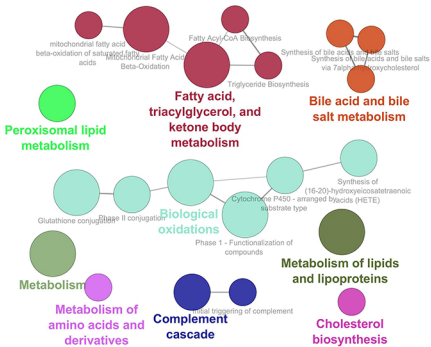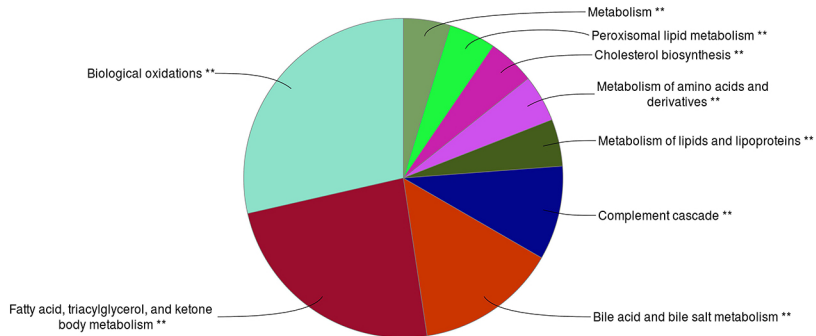

Supplement: Supplementary file 5 — Functional analysis of the “blue” module genes. Over-represented GO/pathway terms were grouped based on kappa statistics [62]. The size of each category within a pie chart represents the number of included terms. Only the most significant GO/ terms within groups were labeled. GO/pathway terms are represented as nodes, and the node size represents the term enrichment significance, while the edges represent significant similarity between categories. (A) Representative biological processes interactions among module genes. (B) Representative molecular function interactions among module genes. (C) Representative Reactome analysis interactions among module genes. (PDF 2680 kb) [file 12865_2017_238_MOESM5_ESM.pdf]

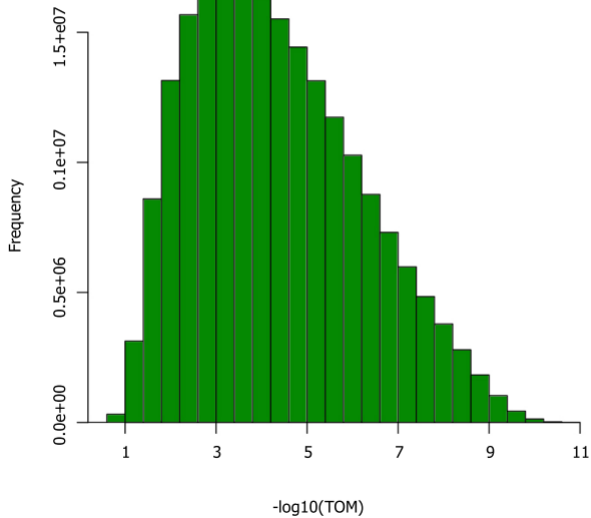

Supplement: Supplementary file 8 — Frequency of TOM connectivity in the network. (PDF 262 kb) [file 12865_2017_238_MOESM8_ESM.pdf]
